# PAR1 Expression Predicts Clinical G-CSF CD34^+^ HSPC Mobilization and Repopulation Potential in Transplanted Patients

**DOI:** 10.1097/HS9.0000000000000288

**Published:** 2019-08-20

**Authors:** Neta Nevo, Tsila Zuckerman, Shiri Gur-Cohen, Orit Kollet, Francesca Avemaria, Elizabeth J. Shpall, Mayela C. Mendt, Arnon Nagler, Benjamin Brenner, Myriam Ben Arush, Tsvee Lapidot

**Affiliations:** 1 The Joan and Sanford Weill pediatric Hematology Oncology and Bone Marrow Transplantation Division, Ruth Rappaport children's Hospital, Haifa, Israel; 2 Department of Immunology, Weizmann Institute of Science, Rehovot, Israel; 3 Hematology and Bone Marrow Transplantation Division, Rambam Health Care Campus, Haifa, Israel; 4 MD Anderson Cancer Center, TX, USA; 5 Hematology and Bone Marrow Transplantation Division, Sheba Medical Center, Tel Hashomer, Israel.

Hematopoietic stem cell transplantation has been established as a curative treatment for patients with hematological malignancies. Understanding the regulation of Hematopoietic Stem and Progenitor Cells (HSPC) is crucial to improve the outcomes of transplants. Others and we have previously shown the role of coagulation-linked pathways in regulation of murine HSPC egress and retention. Particularly, different activities of the major receptor for thrombin, Protease Activated Receptor 1 (PAR1), direct mouse HSPC mobilization versus bone marrow (BM)-retention, driven by thrombin or aPC/EPCR cleavage, respectively. Acute stress and clinical mobilization, upregulate thrombin generation, which cleaves PAR1 to activate pro-inflammatory signaling, inducing HSPC recruitment to the bloodstream. PAR1 can alternatively interact with Endothelial Protein Receptor C (EPCR) activating anti-inflammatory signaling, to retain long term repopulating HSPC in the BM by restricting nitric oxide (NO) generation.^1,2^ These studies investigated the role of coagulation-related pathways only in experimental mouse models and their relevance to clinical protocols is currently unknown.

G-CSF mobilized peripheral blood (PB) cell is a safe source for transplant. However, this procedure results in failure of mobilization in 4% of healthy donors. Factors associated with poor mobilization capacity include low levels of circulating CD34^+^ cells, decreased hematocrit levels and total blood volume, older donor age, white ethnicity and female gender.^3,4^ Better understanding of mechanisms regulating mobilization for stem cell collection and homing for stem cell transplant have therefore high clinical relevance and importance. Taken together, our data suggest that coagulation-related pathways could also be functionally important in regulation of clinical human HSPC mobilization and engraftment kinetics following patient transplantation. In particular, PAR1 expression positively correlates with the mobilization efficiency and engraftment outcome, depicting it as a predictive marker. Importantly, we also show that exposure of human CD34^+^ Cord Blood (CB) cells to a synthetic anticoagulant mimicking peptide^5^ activating aPC/EPCR-mediated PAR1 signaling, specifically improved their homing to the BM of immune deficient NSG mice.

**Figure 1:**
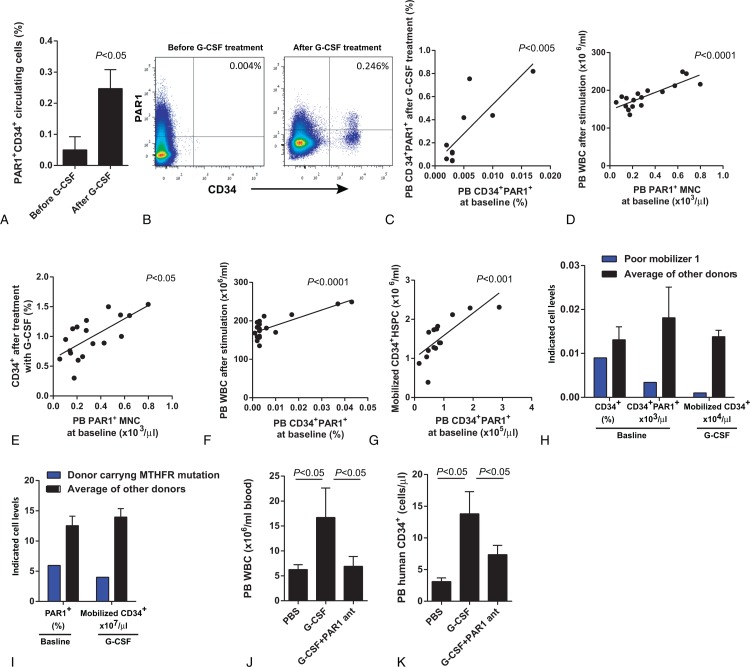
**Expression of PAR1 on circulating MNC positively correlates with G-CSF-induced CD34**^**+**^** HSPC mobilization**. (A) Percent of PAR1 expression levels on circulating CD34^+^ cells before and after treatment with G-CSF. (B) Representative FACS staining for PAR1 and CD34 before and after treatment with G-CSF of one donor out of 20. (C) Correlation between the percentage of PB CD34^+^ PAR1^+^ HSPC before and after treatment with G-CSF. n = 8. PB WBC (D, n = 17) and CD34^+^ HSPC (E, n = 18) after G-CSF treatment vs PB PAR1 expression on MNC at baseline. PB WBC (F, n = 15) and CD34^+^ HSPC (G, n = 14) after G-CSF treatment vs the percentage of PB PAR1 expressing CD34^+^ HSPC and their absolute numbers. (H) The differences of baseline PB CD34^+^ HSPC (%), PAR1 expression on CD34^+^ HSPC and efficiency of mobilization between poor mobilizer donor and the average of other donors (n = 17). (I) The differences of baseline expression of PB PAR1 (%) and efficiency of mobilization between poor mobilizer donor carrying MTHFR mutation and the average of other donors (n = 19). (J) PB WBC following PBS injection (n = 11), or G-CSF treatment with (n = 6) or without (n = 6) administration of PAR1 antagonist in chimeric mice engrafted with human CB MNC. (K) Human PB CD34^+^ cells after treatment with PBS (n = 3), G-CSF (n = 3) or G-CSF and PAR1 antagonist (n = 3) in chimeric mice.

First, we tested if baseline PAR1 expression plays a role in human G-CSF CD34^+^ HSPC mobilization. For this purpose, we chose to analyze the blood of healthy donors in order to avoid possible mobilization failure in the autologous setting. Detailed information about experimental methods is provided in the supplementary methods section. To analyze the role of PAR1 in G-CSF induced mobilization, PB samples were obtained from 20 HSPC donors before and after treatment with G-CSF. We observed high variability of PAR1 expression on circulating CD34^+^ HSPC at baseline among healthy PB stem cell donors, which were up regulated following 5-days treatment with G-CSF (Fig. 1A–B). Interestingly, a strong correlation was found between PAR1 expression on circulating CD34^+^ cells before and after G-CSF treatment (Fig. 1C). Next, we found that baseline PAR1 expression levels on circulating MNC before G-CSF treatment also positively correlated with higher yields of total G-CSF mobilized leukocytes and CD34^+^ HSPC (Fig. 1D–E). In addition, the percentage of PAR1^+^ CD34^+^ HSPC in the blood and their absolute numbers before treatment positively correlated with peripheral blood leukocyte counts (Fig. 1F) and the yield of mobilized CD34^+^ HSPC (Fig. 1G). Accordingly, a poor mobilizer donor (as defined by collection of < 2×10^6^ CD34^+^ cells/kg of recipient body weight), suffering from Ulcerative Colitis, with stable disease under no recent treatment, was characterized by low CD34^+^ cells and PAR1 expression on circulating CD34^+^ cells prior to G-CSF treatment. Importantly, 9 donors with equal or lower CD34^+^ counts but with higher PAR1 expression levels succeeded to better mobilize, highlighting PAR1 as an independent predictor for HSPC mobilization (Fig. 1H). In addition, another poor mobilizer, a thrombophilic donor, carrying the MTHFR mutation, with low PAR1 expression levels on circulating CD34^+^ cells at baseline, had inadequate mobilization following treatment with G-CSF (Fig. 1I). Taken together, these results suggest a role for PAR1 expression in successful human CD34^+^ HSPC mobilization. To assess the need for functional PAR1 signaling in human HSPC mobilization we also utilized chimeric immune-deficient mice, pre-engrafted with human cord blood HSPC. Importantly, blocking PAR1 signaling by in vivo administration of a specific PAR1 antagonist inhibited G-CSF-induced mobilization of human white blood cells (WBC) and CD34^+^ HSPC to the circulation of chimeric mice (Fig. 1J–K).

**Figure 2:**
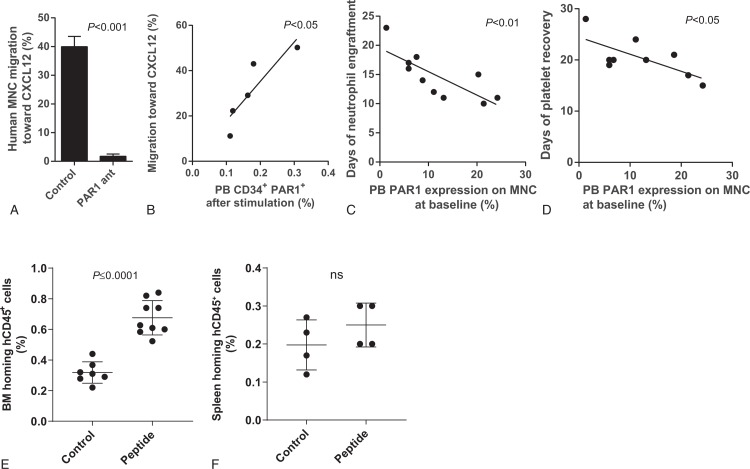
**Impact of PAR1 signaling pathways on migration, engraftment and homing of HSPC**. (A) Human cord blood MNC migration toward CXCL12 1 hour after treatment with PAR1 antagonist (n = 4). (B) MNC migration towards CXCL12 vs. PAR1 expression on PB CD34^+^ cells after treatment with G-CSF. n = 5. Day of neutrophils (C, n = 10) and platelets (D, n = 9) engraftment in transplanted patients vs. expression of PAR1 on donor PB MNC at baseline. (E-F) Human cord blood (CB) CD34^+^enriched cells were incubated with the human aPC/EPCR/PAR1 mimicking peptide for 2hrs prior to their transplantation in pre-clinical model of immune deficient NSG-hSCFTgN mice. Homing levels of human CB CD34^+^ cells to the mouse BM (E) and spleen (F) are shown.

Interactions between CXCL12 and its major receptor CXCR4 play a major role in human and murine G-CSF-induced HSPC mobilization.^4,6,7^ We, therefore, expected PAR1 inhibition to affect also HSPC migration. Blocking PAR1 signaling by the specific PAR1 antagonist also inhibited in vitro migration of human BM or cord blood MNC towards a gradient of CXCL12, (Fig. 2A) similar to murine BM MNC migration.^1^ Importantly, PAR1 expression on mobilized CD34^+^ cells correlated with mobilized MNC migration towards a gradient of CXCL12 (Fig. 2B). PAR1 regulates endothelial nitric oxide synthase (eNOS) which induces NO production.^8^ In line with our results, NO donor treatment upregulated surface CXCR4 expression on human cord blood HSC, promoting their in vitro CXCL12 induced chemotactic response, and in vivo homing and engraftment of immune deficient NSG mice.^9,10^ Importantly, in CD34^+^ stem and progenitor cells mobilized by G-CSF treatment in healthy donors, a positive correlation between CXCR4 expression, CXCL12-induced migration and both stem cell mobilization efficiency and patient engraftment was reported.^11^ To evaluate the role of PAR1 in BM engraftment, we followed recovery parameters of patients transplanted with the G-CSF-mobilized cells. Importantly, we found accelerated neutrophil and platelet engraftment in patients transplanted with mobilized cells expressing higher PAR1 levels on MNC at baseline (Fig. 2C–D). These correlations suggest PAR1 expression before G-CSF treatment in donors as a predictive marker for efficiency of patient blood count recovery as well.

After their infusion, HSPC migrate in the circulation and home to the recipient BM, where they lodge and reconstitute the BM with normal blood and immune cells. This process of migration and BM recognition is termed homing and is an essential first step for successful transplantation. Since the late 80’, CB transplants (CBT) has emerged as an alternative source of HPSC for HCT.^12^ CB offers the advantage of easy and quick procurement, the absence of risk to the donors, reduced risk of transmitting infections and a lower risk of Graft vs Host Disease (GVHD) thus permitting less stringent HLA matching. The major disadvantage of CBT is the late hematopoietic recovery, which leads to increased early morbidity and mortality after transplantation.^13^ In order to rapidly reconstitute the BM, blood and immune system of transplanted patients there is a need for increased number of stem and progenitor cells, according to the size and body weight of the patients. Delayed neutrophil and platelet engraftment with CB is associated with significant infection and bleeding related morbidity/mortality limiting its widespread use.

To overcome this risk, we designed to target to the BM not only EPCR-expressing HSPC but also short-term CD34^+^ progenitors and maturing leukocytes which express PAR1 but have reduced levels or lack expression of EPCR. These cells are important to provide the short-term engrafting leukocytes, which are essential for neutrophil and platelet recovery. As PAR1 blockade inhibited migration, we aimed to influence CB homing in vivo, by activating the alternative PAR1 pathway leading to cytoprotective and anti-inflammatory effects.^5^ For this purpose, we synthesized a peptide mimicking the PAR1 fragment naturally cleaved by aPC/EPCR.^5^ Importantly, we found that a short (2 h) pretreatment of human CB CD34^+^ enriched cells before their transplantation into immune deficient mice significantly elevated their homing levels specifically to the BM but not to the spleen (Fig. 2E–F).

To conclude, we reveal for the first time PAR1 expression levels on circulating MNC and CD34^+^ cells as new independent parameters for predicting both the efficiency of human PB CD34^+^ HSPC mobilization by G-CSF and hematological recovery of patients, after matched allogeneic transplantation. Although the sample size in the study is small, still the correlations we have found are constant, statistically significant and may stimulate new insight regarding the involvement of the coagulation system in clinical human G-CSF induced mobilization of stem and progenitor cells. In line, our research is supported by a previous study reporting that PAR1 levels genetically assessed by microarrays were found to be 3.3-fold higher in G-CSF-mobilized human CD34^+^ HSPCs compared with steady-state BM CD34^+^ HSPC.^14^

To improve CBT a combination of pro-engraftments approaches involving ex vivo HSPC expansion and modulation of HSPC functionality have been studied extensively in animal models and clinical trials.^15^ Here, we present a new approach to enhance homing of CD34^+^ CB cells via exposure of these cells to a synthetic peptide activating anti-inflammatory coagulation-related signaling.

Our study therefore adds important clinically relevant understanding of mechanisms regulating human HSPC mobilization and transplantation. In addition, our results are promising as to the concept that HSPC homing can be improved by pre-stimulation of cells prior to their transplantation. Here we identified a new player participating in regulation of human HSPC, with potential to predict efficiency as well as clinical outcome of G-CSF-induced mobilization, homing, and engraftment kinetics as well as efficiency. Better understanding of HSPC regulation may help improving G-CSF mobilization in autologous setting, CBT engraftment, haplo-transplantations and transplantation for myelofibrosis. Future studies will reveal whether manipulations of PAR1 expression and function in human HSPC can improve the efficiency of these clinical procedures.
